# Effects of prebiotics from diverse sources on dysbiotic gut microbiota associated to western diet: Insights from the human Mucosal ARtificial COLon (M-ARCOL)

**DOI:** 10.1016/j.crfs.2024.100968

**Published:** 2024-12-26

**Authors:** Ophélie Uriot, Clémence Defois-Fraysse, Ingrid Couturier, Charlotte Deschamps, Claude Durif, Cyril Chaudemanche, Assia Dreux-Zigha, Stéphanie Blanquet-Diot

**Affiliations:** a UMR 454 MEDIS, Microbiologie Environnement Digestif et Santé, Université Clermont Auvergne – INRAE, Clermont-Ferrand, France; b Greencell, Biopôle Clermont Limagne, Saint-Beauzire, France; c General Mills France, Boulogne-Billancourt, France

**Keywords:** *In vitro* human gut model, intestinal microbiota, mucus, western diet, inulin, RFO, laminarin

## Abstract

Associated to various illnesses, Western Diet (WD) is acknowledged to have deleterious effects on human gut microbiota, decreasing bacterial diversity, lowering gut bacteria associated to health (such as *Akkermansia muciniphila)*, while increasing those linked to diseases (e.g., *Proteobacteria*). In this study, we evaluated the potential of two new prebiotics to counteract the negative effect of WD on gut microbiota, namely raffinose family oligosaccharides (RFO) from chickpeas and laminarin (LAM) from algae, when compared to the well-known inulin (INU). The effects of prebiotics on gut microbiota composition and metabolic activities were investigated in the Mucosal-Artificial Colon, set-up to reproduce WD condition, as compared to healthy control (n = 3). None of the prebiotics was able to efficiently offset the shift in microbiota induced by WD. Nevertheless, when compared to non-supplemented WD, all prebiotics showed significant impacts on microbiota composition, that were both prebiotic and donor-dependant. RFO was the only prebiotic to enhance α-diversity, while it led to an increase in *Blautia* and *Butyricicoccaceae*, associated with higher amounts of gas and butyrate. LAM and INU did not strongly impact microbial metabolic activities but were associated with a rise in *Prevotella_9*/*Agathobacter* and *Faecalibacterium,* respectively. To conclude, this study showed that all tested prebiotics had different impacts on human gut microbiota structure and activities, which was further donor-dependent. M-ARCOL appears as a suitable *in vitro* tool to better understand the mechanisms of action of prebiotic compounds in relation to gut microbes and define responders and non-responders to prebiotic supplementation, opening the possibility of customized nutritional strategies.

## Highlights

• All prebiotics differently modulated gut microbiota composition.
• Raffinose Family Oligosaccharides increase alpha-diversity compared to western diet.
• Raffinose Family Oligosaccharides supplementation increase butyrate production.
• Laminarin and inulin did not strongly impact microbial metabolic activities.
• Donor-dependent responses of gut microbiota to prebiotic treatments.

## Graphical abstract

**Figure:**
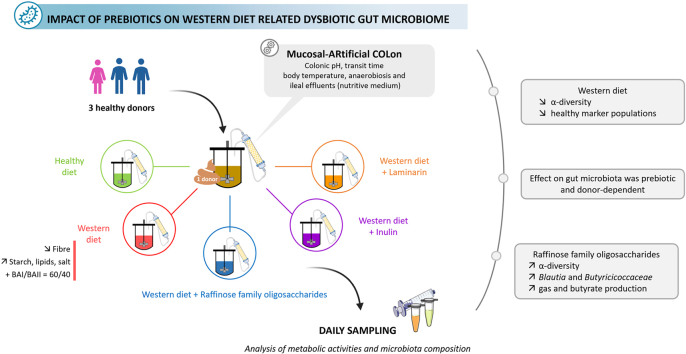

## 1 Introduction

The human digestive tract hosts a complex community of microorganisms (Thursby and Juge, 2017), referred to as the intestinal microbiota, which plays a key role in host nutrition, immunity and health (Thursby and Juge, 2017; Martin et al., 2019). Many internal and external factors are shaping gut microbes, one of the main being human diet (Thursby and Juge, 2017; Zhang, 2022). Western diet (WD), defined as low in fibre and high in fat and sugar (Clemente-Suárez et al., 2023), is well known to have a deleterious effect on the gut microbiota through decreasing bacterial diversity and impacting microbial composition. WD reduces gut bacteria associated to health such as *Bifidobacterium* spp., *Akkermansia muciniphila*, and increases those associated to health deleterious effect like *Proteobacteria*, *Oscillibacter* spp. and *Desulfovibrio* spp. related to intestinal barrier dysfunction (Clemente-Suárez et al., 2023; Hills et al., 2019; Suriano et al., 2022; Zhang and Yang, 2016). Associated to a sedentary lifestyle, WD promotes diseases such as obesity, type 2 diabetes mellitus, dyslipidaemia, inflammatory bowel disease, neoplasms, and cardiovascular diseases (Clemente-Suárez et al., 2023; Malesza et al., 2021). Many nutritional or pharmaceutical strategies are currently studied to rebalance WD dysbiotic microbiota towards a healthier state and thus restore its protective activities to the host (Hills et al., 2019; Martinez et al., 2017). One of the most relevant strategies is the use of prebiotic, recently redefined as “a substrate that is selectively utilized by host microorganisms conferring a health benefit” (Gibson et al., 2017). It is universally acknowledged that inulin as a prebiotic has an outstanding effect on the regulation of intestinal microbiota and exhibits many health benefits in improving constipation, regulating lipid metabolism, weight loss, lowering blood sugar, enhancing mineral absorption and reducing the risk of colon cancer (Qin et al., 2023).

The potential of other prebiotics extracted from vegetables, legumes or algae is currently under investigation in the context of human health (Bamigbade et al., 2022; Cherry et al., 2019). Raffinose family oligosaccharides (RFO), present in most legumes, are composed of ꭤ-(1–6) galactosides linked to one sucrose (Cardoso et al., 2021; Elango et al., 2022). Previous studies in rodents or humans have shown that RFO promotes abundances of beneficial species in stool samples such as *Bifidobacteria*, *Lactobacilli* or butyrate-producing bacteria (e.g., *Faecalibacterium prausnitzii*) and reduces the presence of *Proteobacteria* (Elango et al., 2022; Amorim et al., 2020; Dai et al., 2019; Fernando et al., 2010). Most probably in relation with those changes, RFO had shown benefits on human health, such as anti-allergic, anti-obesity and anti-diabetic effects and would participate in the prevention of non-alcoholic fatty liver disease by inhibiting lipid accumulation (Elango et al., 2022; Dai et al., 2019; Moghaddam et al., 2018; Muthukumaran et al., 2018; Nagura et al., 2002; Shakappa et al., 2018; Watanabe et al., 2004). However, since RFO may be responsible for flatulence and digestive discomfort (Elango et al., 2022), it is important to find the right balance between concentration and effects and evaluate their use in sensitive people. Another sustainable and renewable source for prebiotics is algae (Cherry et al., 2019; Shannon et al., 2021). Laminarin, extracted from brown algae, is a complex polysaccharide composed of β(1–3)-linked glucose units with β(1–6)-branches (Shannon et al., 2021). Evidences on health benefits of laminarin have been yet obtained only in animal models, with anti-obesity, anti-inflammatory, anti-cancer and antioxidant effects (Shannon et al., 2021; Nguyen et al., 2016; Song et al., 2017; Zhang et al., 2023). In particular, in pigs, laminarin promoted intestinal lactobacilli and *Prevotella* together with a reduction in *Enterobacteriaceae*, showed anti-inflammatory effects and prevented post-weaning intestinal dysfunction (Zhang et al., 2023; Rattigan et al., 2020; Vigors et al., 2020). In mice fed with high-fat diet, supplementation with laminarin led to an increase in Bacteroidetes while Firmicutes decreased, associated with a slower weight gain (Nguyen et al., 2016).

Up to now, most studies evaluating the interactions of RFO or laminarin and gut microbes have been performed in animals or based on human stool samples analysis. It would be of great interest to evaluate those connections in the human colonic compartment, hosting different microbes and showing specific metabolic activities. For ethical, regulatory and cost reasons, *in vitro* models simulating the human large intestinal compartment can be used as an alternative to *in vivo* assays, especially to perform mechanistic studies on gut microbiome. Among those *in vitro* models, the most dynamic and complex are able to reproduce the main nutritional (availability of nutrients), physicochemical (pH, temperature, transit time, anaerobiosis) and microbial (lumen and mucus-associated microorganisms) parameters found in the human colon, such as the Mucosal Simulator of Human Intestinal Microbial Ecosystem (Lambrecht et al., 2021; Marzorati et al., 2020) or the Mucosal ARtificial COLon (M-ARCOL) (Deschamps et al., 2020; O'Sullivan et al., 2024).

In this context, the aim of this study was to investigate the effect of RFO and laminarin on human colonic microbiota composition and functions under WD-related dysbiosis, as compared with the well-known prebiotic inulin. Continuous fermentations were carried out during 14 days in the M-ARCOL model, fed with a high fat-high sugar WD, and supplemented with 2.5 g/day of RFO, laminarin or inulin. Microbiota composition and metabolic activities were followed by 16S Metabarcoding and short-chain fatty acids (SCFA) and gas measurements, respectively. Results were compared with two control conditions, *i.e.*, non-supplemented under WD and non-supplemented under healthy diet.

## 2 Materials and methods

### 2.1 Prebiotics sourcing and extraction

This study included three prebiotics. An agave-derived powdered long-chain inulin from General Mills France (Boulogne-Billancourt, France) was used as a prebiotic control. Inulin powder contained fructose polymers (>90 % dry weight) with minor amounts of fructose (<12 % dry weight), sucrose and glucose. Laminarin extract from *Saccharina latissima* algae and RFO extract from *Cicer arietinum* chickpeas were provided by our partner GREENCELL (Biopôle Clermont-Limagne, Saint-Beauzire, France). Briefly, the *Saccharina latissima* algae underwent an acid hydrolysis followed by a 50 kDa ultrafiltration and a precipitation with ethanol 96 % overnight. The precipitate was then dried in an oven to obtain the laminarin extract (Laminarin MW 5 kDa). The *Cicer arietinum* chickpeas were crushed and underwent ethanolic extraction followed by a 5 kDa ultrafiltration. The permeate was then concentrated and freeze-dried to obtain the RFO extract (RFO MW 0.5–0.8 kDa). The extracts were characterised by their prebiotic content (RFO: 20 % and laminarin: 44 %).

### 2.2 Faeces origin and treatment

**Fig. 1:**
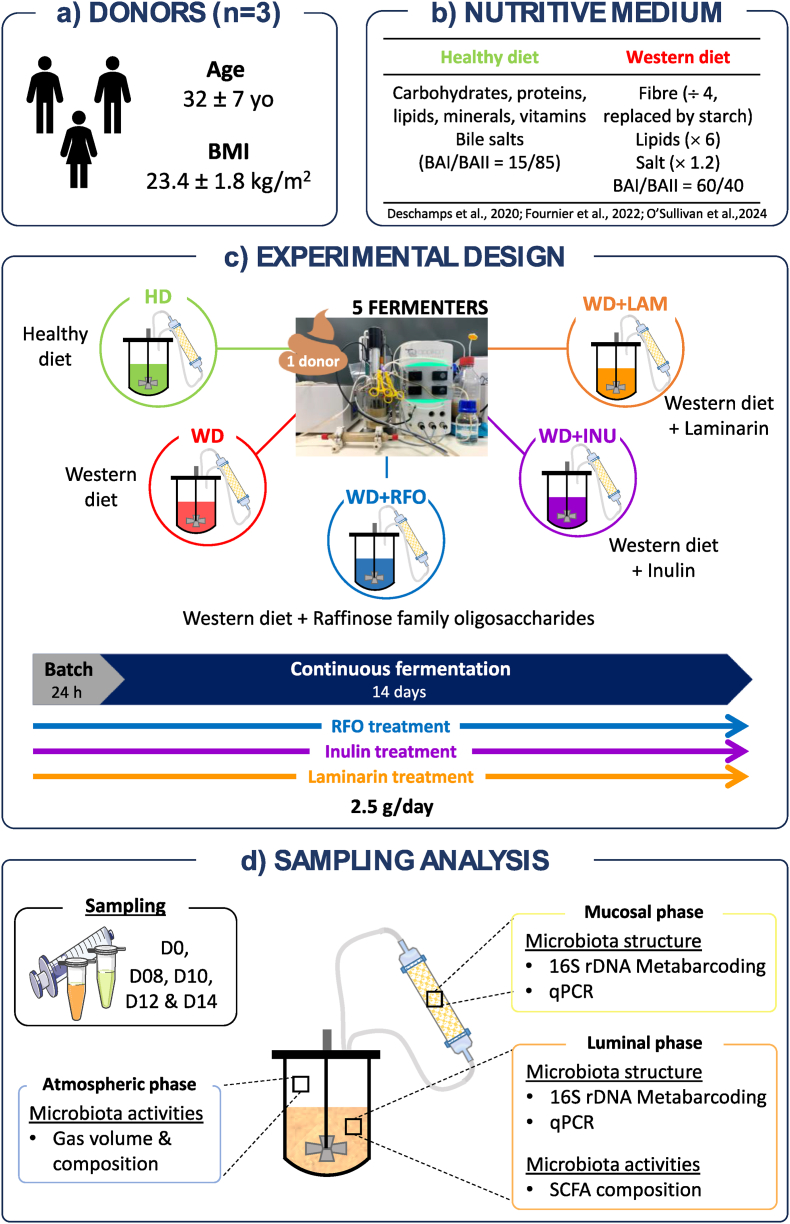
Experimental design, sampling and analysis in the M-ARCOL (a) The M-ARCOL model was inoculated with faecal sample from three healthy volunteers (one women and two men corresponding to three biological replicates). (b–**c**) Five bioreactors were run in parallel for 15 days to test five different conditions, *i.e.*, healthy diet (HD), western diet (WD), western diet supplemented with raffinose family oligosaccharides (WD + RFO), western diet supplemented with inulin (WD + INU) and western diet supplemented with laminarin (WD + LAM). (d) Samples were regularly collected in the luminal and mucosal compartments as well as the atmospheric phase of bioreactors to monitor microbiota composition and metabolic activities. BAI: primary bile acids, BAII: secondary bile acids, BMI: body mass index, HD: healthy diet, INU: inulin, LAM: laminarin, RFO: raffinose family oligosaccharides, SCFA: short-chain fatty acids, WD: western diet.

Fresh faecal samples were collected from three healthy adult volunteers (2 men, 1 woman, 32 ± 7 yo, BMI: 23.4 ± 1.8 kg/m^2^, Fig. 1) with no history of antibiotic nor prebiotic or probiotic supplementation two months prior to the beginning of the study. No specific dietary survey was carried out prior stool donation, however it was determined that the donors had a Mediterranean-type diet, except for Donor_2 who exhibited a Western diet pattern. This study was a noninterventional study with no additions to usual clinical care. Stool samples were collected and kept anaerobically for a maximum of 12 h before inoculation. Then, they were processed under strict anaerobic conditions (COY Laboratory Products Inc, Grass Lake, MI, USA) before their introduction into the M-ARCOL system. Each stool was tenfold diluted into 30 mM anaerobic sodium phosphate buffer (pH 6.5) supplemented with 1.9 mM cysteine, then filtered through a 500 μm sieve.

### 2.3 M-ARCOL system

The M-ARCOL system is a one-stage fermentation system (Fig. 1) used under continuous conditions which simulates, based on *in vivo* data, the main physicochemical (pH, body temperature, retention time), nutritional (composition of ileal effluents used as a nutritive medium) and microbial (lumen and mucus-associated microbiota) conditions found in the human colon (Deschamps et al., 2020; O'Sullivan et al., 2024; Etienne-Mesmin et al., 2022; Fournier et al., 2022). At the beginning of the experiments, each glass vessel was set under anaerobiosis by flushing with O_2_-free N_2_ gas. Then, the bioreactors were inoculated with 200 mL of nutritive medium and 100 mL of the faecal suspension. Throughout fermentation, anaerobiosis was maintained by the sole activity of the resident microbiota and by ensuring the system airtightness (Deschamps et al., 2020). Bioreactors were kept at body temperature using an incorporated heating block. Colonic pH was set and maintained at 6.3 by automatic addition of 2 M NaOH. The redox potential was also constantly recorded (Applikon, Delft, The Netherlands). Nutritive medium containing various sources of carbohydrates, proteins, lipids, minerals, and vitamins was continuously introduced into the bioreactors, while the fermentation medium was automatically withdrawn, ensuring the appropriate retention time of 24 h inside the bioreactors (which can be correlated with a total colonic transit time of 48 h, in agreement with *in vivo* data found in human adults). Overproduced gases were collected daily in a sampling bag connected to the luminal vessel. A second airtight vessel containing mucin-alginate beads (5 % of mucin from porcine stomach type II, and 2 % of sodium alginate both from Sigma-Aldrich, Saint-Louis, MO, USA) allowed to differentiate lumen- and mucus-associated microbiota (Deschamps et al., 2020; O'Sullivan et al., 2024).

### 2.4 Experimental design

Each stool (n = 3) was used to inoculate five bioreactors ran in parallel (Fig. 1), one fed with healthy diet (HD) (Deschamps et al., 2020; Fournier et al., 2022) and the four others with WD (O'Sullivan et al., 2024), *i.e.*, depleted in fibre and enriched in lipids, starch, salts and primary bile acids (Fig. 1b). One among those four bioreactors was non-supplemented (WD) while the three others received 2.5 g/day of prebiotic, namely inulin (WD + INU), RFO (WD + RFO) and laminarin (WD + LAM). After a 24 h batch fermentation allowing microbiota amplification, bioreactors were run under continuous conditions during 14 additional days. Samples were daily collected from the main vessel (termed luminal compartment) and centrifugated (18,000 g, 15 min, 4 °C). Pellets were kept at −80 °C for downstream analysis of luminal gut microbiota and supernatants were collected and stored at −20 °C for further SCFA measurement. Atmospheric phase was also sampled every day to check anaerobic condition and determine gas composition. The total volume of overproduced gases was measured daily. Every two days, mucin beads were collected from external vessel (termed mucosal compartment) and renewed by fresh ones under a CO_2_-flow. Beads were washed twice in sterile phosphate buffer saline and stored at −80 °C before microbiota analysis.

### 2.5 DNA extraction

Genomic DNA was extracted from luminal and mucosal samples using the QIAamp Fast DNA Stool Mini Kit® (Qiagen, Hilden, Germany). Samples were incubated 10 min at 37 °C with 2 mL of 55 mM citrate buffer, then vortexed 3 min at full speed (Capone et al., 2013). After centrifugation (8,000 g, 1 min, 20 °C), pellets were subjected to mechanical disruption using a bead beater (5 min, 20 beat/s) with 300 mg of sterile glass beads (diameter ranging from 0.1 to 0.6 mm) and 1 mL of InhibitEX® buffer from Qiagen kit. Then, DNA was extracted following manufacturer's instructions. DNA quantity was measured with the Qubit dsDNA Broad Range Assay Kit® (Invitrogen, Carlsbad, CA, USA) on a Qubit 3.0 Fluorometer® (Invitrogen, Carlsbad, CA, USA). DNAs were stored at −20 °C prior to quantitative PCR analysis and 16S Illumina sequencing.

### 2.6 qPCR: total bacteria quantification

Total bacteria from luminal and mucosal samples of the M-ARCOL were quantified by qPCR using the primers BAC338R (5′-ACTCCTACGGGAGGCAG-3′) and BAC516F (5′-GTATTACCGCGGCTGCTG-3′) with a hybridization temperature set at 58 °C (Yu et al., 2005). Real-time qPCR was performed on a Biorad CFX96TM Real-Time System (Bio-Rad Laboratories, Hercules, USA) using Takyon Low ROX SYBR 2X MasterMix blue dTTP kit (Eurogentec, Liège, Belgium). Each reaction was run in duplicate in a final volume of 10 μL with 5 μL of Master Mix, 0.45 μL of each primer (10 μM), 3.1 μL of ultra‐pure water and 1 μL of DNA sample or 1 μL of ultra-pure water for negative control. The amplification conditions consisted in 1 cycle at 95 °C for 5 min, followed by 40 cycles of 95 °C for 30 s, 58 °C for 30 s and 72 °C for 30 s. A melting step was added to ensure primer specificity. Standard curve was generated from 10-fold dilutions of bacterial DNA (extracted from a luminal medium sample), allowing the calculation of DNA concentrations from extracted samples (Fournier et al., 2023).

### 2.7 16S rDNA Metabarcoding and data analysis

The V3-V4 region of 16S ribosomal DNA (rDNA) was amplified using V3_F357_N (5′-CCTACGGGNGGCWGCAG-3′) and V4_R805 (5′-GACTACHVGGGTATCTAATCC-3′) (Klindworth et al., 2013). Amplicons were generated using a Fluidigm Access Array followed by high-throughput sequencing on an Illumina MiSeq system (Illumina, San Diego, CA, USA) performed at the Carver Biotechnology Center of the University of Illinois (Urbana Champaign, IL, USA). Prior to analysis, raw data were demultiplexed and quality filtered using ‘DADA2’ R-package (Callahan et al., 2016). Reads with quality score under 2 were truncated. Reads under 100 pb length were removed as well as sequences similar to PhiX DNA used as a spike-in control for MiSeq runs. Filtered sequences were dereplicated and cleaned for chimeras (DADA2). Taxonomic classification of the sequences was then performed with ‘DECIPHER’ package (Murali et al., 2018). Assignations from both SILVA 138 release (Quast et al., 2013) and GTDB_bac120_arc122 (Parks et al., 2022) databases were merged using rANOMALY R-package based on IDTAXA, with a 60 % confidence cut-off. A phylogenetic tree was then reconstructed using functions from phangorn package (Schliep, 2011). Incorrect taxonomic affiliations were manually corrected after verification in NCBI; *Prevotella* genus was affiliated with *Prevotellaceae, Ruminococcus* genus with *Ruminococcaceae* family, the *Clostridium* genus with *Clostridiaceae* family.

### 2.8 Gas analysis

Relative abundances of O_2_, N_2_, CO_2_, CH_4_, and H_2_ present in the fermenter atmospheric phase was determined using a 490 micro‐gas chromatography (Agilent Technologies, Santa Clara, CA, USA) equipped with Molecular Sieve 5A column and PoraPlot U column coupled with thermal conductivity detector TCD detectors. Argon was used as the carrier gas. Calibration curves were made from four gases: (i) ambient air (78 % N_2_, 21 % O_2_ and 0.04 % CO_2_), (ii) mixture A (5 % CO_2_, 5 % H_2_ and 90 % N_2_), (iii) mixture B (20 % CO_2_ and 80 % H_2_), and mixture C (19.9 % CO_2_, 19.9 % CH_4_, 20 % H_2_ and 40 % N_2_). Results were expressed as relative percentages.

### 2.9 SCFA analysis

For SCFA analysis, 1.5 mL of luminal medium samples were centrifuged (18,000 g, 15 min, 4 °C) and 900 μL of supernatant was diluted with 100 μL of H_2_SO_4_ 0.04 M mobile phase, vortexed and filtered (0.22 μm). Concentration of the three main SCFA (acetate, propionate and butyrate) were determined in the luminal samples using high performance liquid chromatography (HPLC) (Elite LaChrom, HITACHI, San Jose, CA, USA) coupled with a diode‐array detector. The HPLC column (Concise Separations, San Jose, CA, USA) was maintained at 50 °C. A mobile phase composed of sulfuric acid 0.04 M was used at a flow rate of 0.6 mL/min to separate the different SCFAs. Data were analysed by the EZChrom Elite software at 205 nm. SCFA concentrations (expressed as mM or relative percentages) were calculated from standard curves established with known concentrations of acetate, propionate and butyrate (0, 10, 25 and 40 mM).

### 2.10 Statistical analysis

Statistical analyses on microbiota activities (gases and SCFA), alpha-diversity indexes (number of observed ASVs and Shannon index) from 16S Metabarcoding were processed using R studio version 4.3.1 and with the application of Wilcoxon test. Beta-diversity was evaluated by principal coordinate analysis (PCoA) and constraint redundancy analysis (RDA) was performed with time, treatment, micro-environment (*i.e.* luminal and mucosal), and donor as variables, first with all parameters and then with removal of the donor variable using VEGAN package (Oksanen et al., 2019). Bray–Curtis distances were used for each analysis, and significance between the groups was assessed with one- or two-way ANOVA. Differential analyses were performed using three methods: DESeq2, metagenomeSeq and metacoder from rANOMALY v1.0.0 package (Theil and Rifa, 2021). Spearman correlations analyses between metabolic variables and bacterial genera were performed and associated heatmaps were generated using the ‘microeco’ package (Liu et al., 2021a).

## 3 Results

### 3.1 Characterisation of stool samples used for M-ARCOL inoculation and microbiota stabilisation in the *in vitro* model

**Fig. 2:**
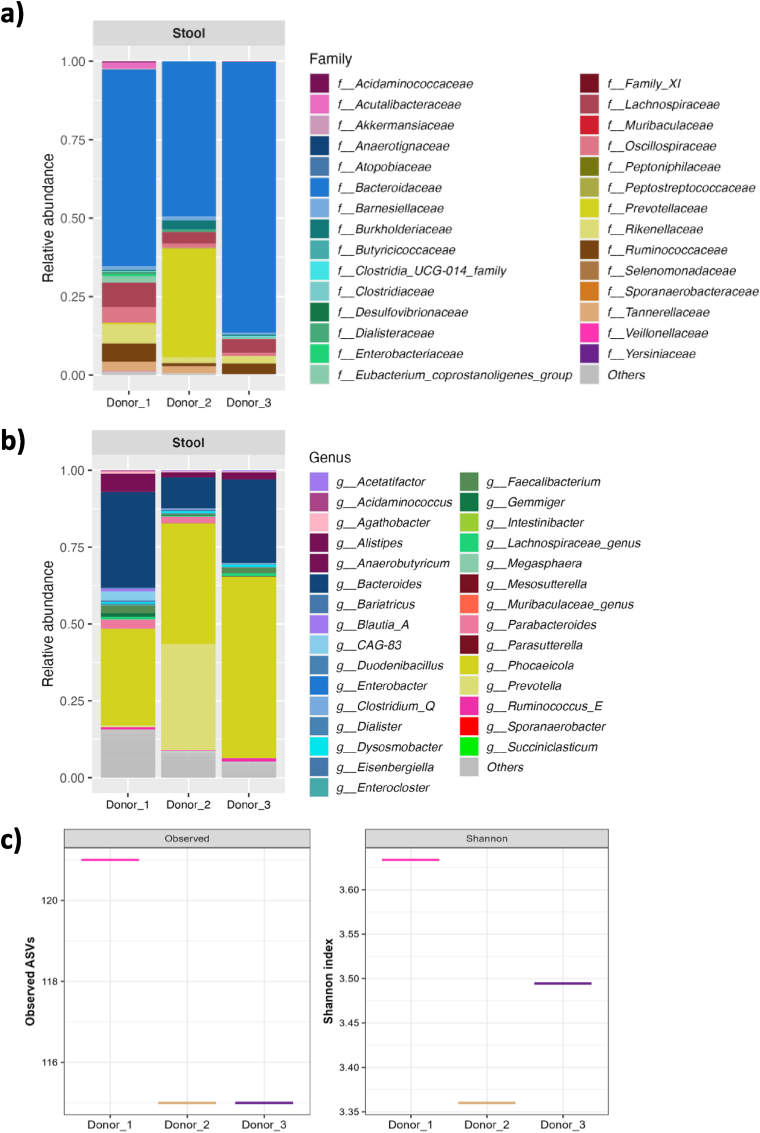
Characterisation of stool samples used for M-ARCOL inoculation Stool samples were collected from three healthy volunteers (Donor_1, 2 and 3). Microbiota composition was analysed by 16S rRNA Metabarcoding. Bacterial abundances are represented at the family (**a**) and genus (**b**) levels for each donor. Alpha-diversity represented by number of observed ASVs and Shannon indexes was also calculated for each individual (**c**).

M-ARCOL was used to assess the impact of three prebiotics on human colonic microbiota. The faecal samples from three healthy adult donors were used to inoculate bioreactors (Fig. 1) and characterised in terms of microbiota composition and metabolic activities (Fig. 2). Faecal inoculates showed different bacterial profiles at the family level (Fig. 2a), even more pronounced at a lower taxonomic level (Fig. 2b). Briefly, at the family level, Donor_1 was characterised by the higher relative abundances of *Acutalibacteraceae*, *Akkermansiaceae*, *Lachnospiraceae*, *Oscillospiraceae*, *Rikenellaceae* and *Ruminococcaceae*, while Donor_2 had the higher prevalence of *Prevotellaceae* and the lowest level of *Bacteroidaceae* and *Ruminococcaceae* and Donor_3 was characterised by the greatest amount of *Bacteroidaceae.* Faecal samples also exhibited different bacterial α-diversity, Donor_1 showing the greatest number of observed ASVs (121 *versus* 115 for Donor_2 and 3) and the highest Shannon index (Fig. 2c). Main SCFA were also quantified in faecal samples, but concentrations were below the detection limit (data not shown).

**Fig. 3:**
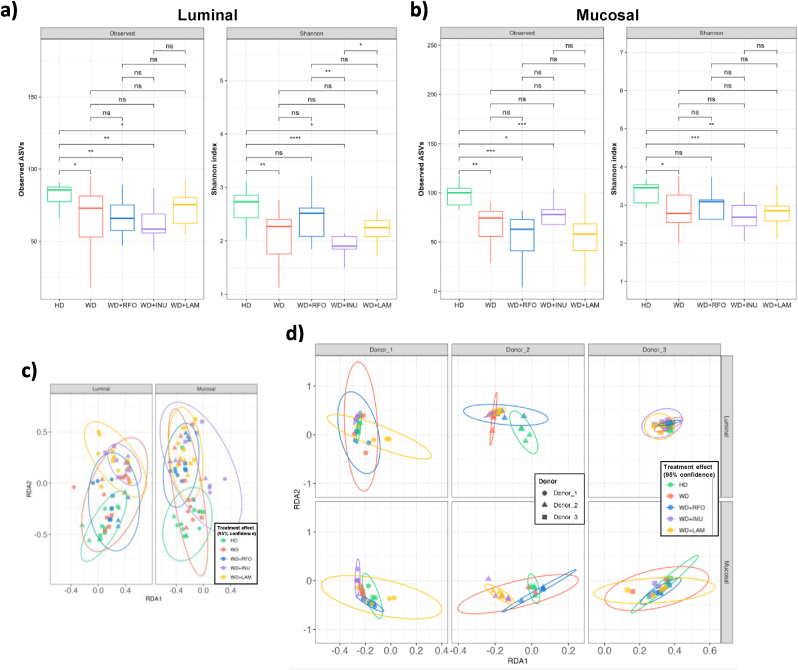
Impact of prebiotic treatment on bacterial diversity in the M-ARCOL Fermentations were performed in the M-ARCOL under five different conditions, *i.e.*, healthy diet (HD), western diet (WD), western diet supplemented with RFO (WD + RFO), western diet supplemented with inulin (WD + INU) and western diet supplemented with laminarin (WD + LAM), after inoculation with stools from healthy adult volunteers (n = 3). Lumen and mucus-associated microbiota composition was analysed by 16S rRNA Metabarcoding and diversity indexes were calculated based on ASV table. Results obtained from days 8–14 are represented. Alpha-diversity indexes (number of observed ASVs and Shannon index) are given as box plots in the luminal (**a**) and mucosal (**b**) compartments. Redundancy analysis (RDA) two-dimension plot visualization of bacterial diversity showed the effect of prebiotic treatments when removing the donor effect (**c**) or by donors (**d**). Significant differences based on Wilcoxon test are presented as ∗p<0.05, ∗∗p<0.01, ∗∗∗p<0.001 and ∗∗∗∗p<0.0001. ns: non-significant difference (p>0.05). HD: healthy diet, INU: inulin, LAM: laminarin, RFO: raffinose family oligosaccharides, WD: western diet.

**Fig. 4:**
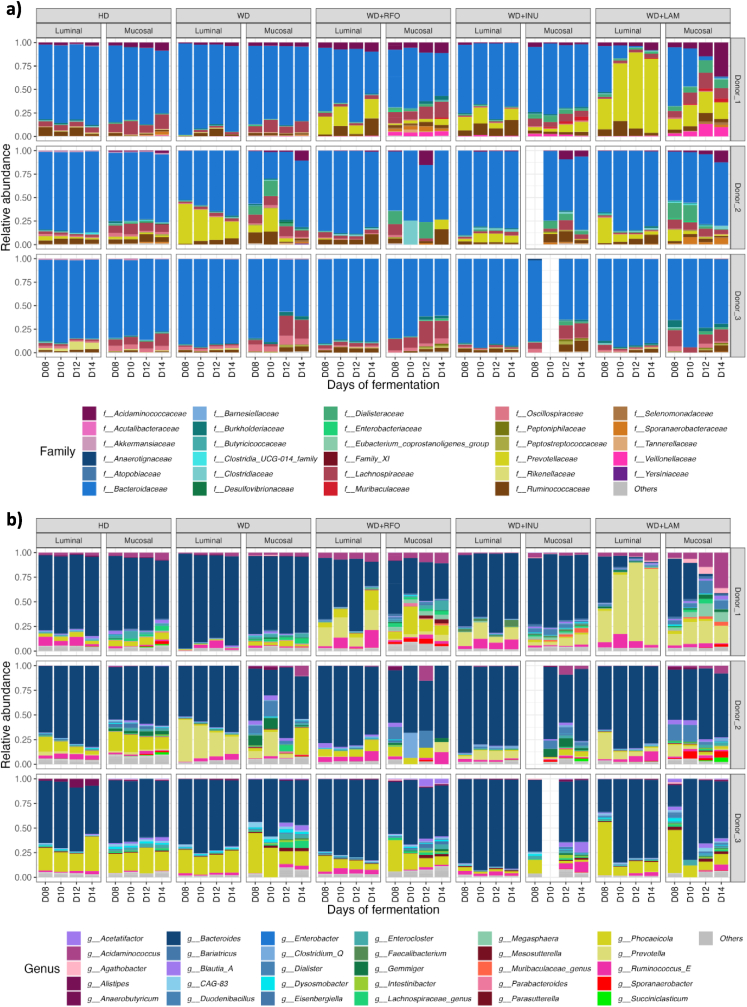
Impact of prebiotic treatments on bacterial composition in the M-ARCOL Fermentations were performed in the M-ARCOL under five different conditions, *i.e.*, healthy diet (HD), western diet (WD), western diet supplemented with RFO (WD + RFO), western diet supplemented with inulin (WD + INU) and western diet supplemented with laminarin (WD + LAM), after inoculation with stools from healthy adult volunteers (n = 3). Lumen and mucus-associated microbiota composition was analysed by 16S rRNA Metabarcoding. Relative abundance of the main bacterial populations in both colonic microenvironments are represented only for stabilised days (days 8–14) at the family (**a**) and genus (**b**) levels. HD: healthy diet, INU: inulin, LAM: laminarin, RFO: raffinose family oligosaccharides, WD: western diet.

**Fig. 5:**
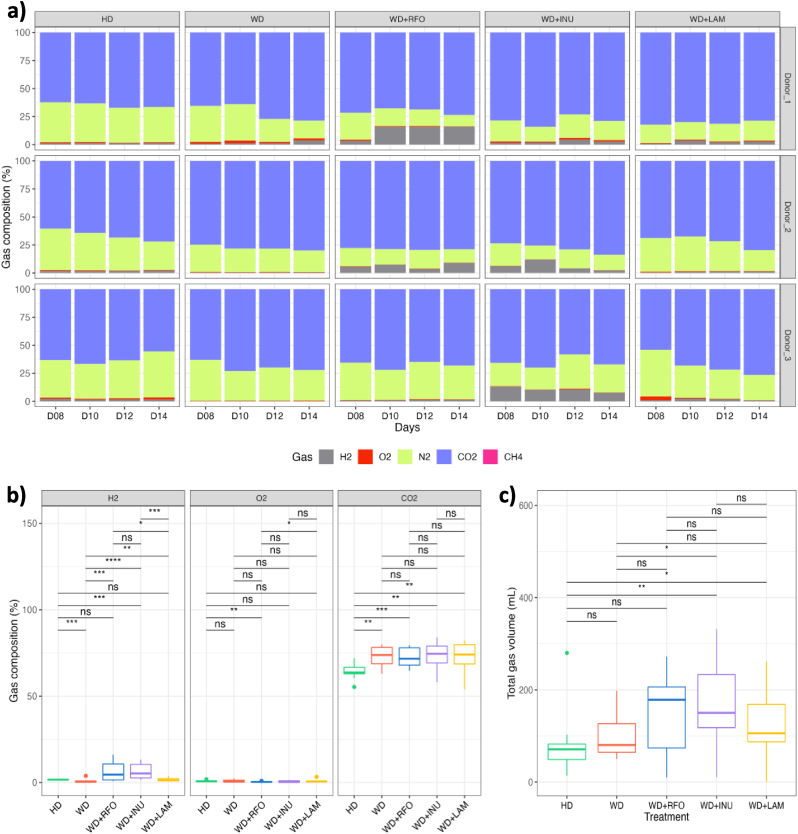
Impact of prebiotic treatment on atmospheric gases in the M-ARCOL Samples were regularly collected from the atmospheric phase of M-ARCOL bioreactors to determine gas composition. Results are expressed in relative percentages for each donor from day 8 to day 14 (**a**). H_2_, O_2_ and CO_2_ relative percentages were averaged from days 8–14 and represented per condition (**b**). Mean daily gas volume is given in mL (**c**). Significant differences based on Wilcoxon test are presented as ∗p<0.05, ∗∗p<0.01, ∗∗∗p<0.001 and ∗∗∗∗p<0.0001. ns: non-significant difference (p>0.05). HD: healthy diet, INU: inulin, LAM: laminarin, RFO: raffinose family oligosaccharides, WD: western diet.

**Fig. 6:**
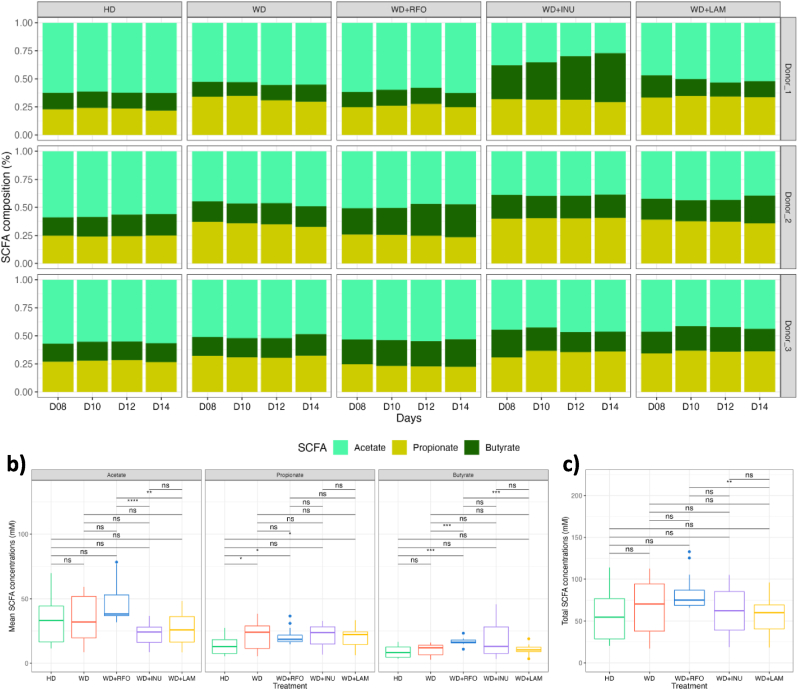
Impact of prebiotic treatments on short-chain fatty acids production Samples were regularly collected from the luminal medium of the M-ARCOL bioreactors to determine short-chain fatty acids (SCFA) concentrations. The three main SCFA (*i.e.*, acetate, propionate and butyrate) were measured daily and results obtained from day 8 to day 14 are expressed as mean relative percentages for each donor (**a**). Results obtained from even days 8–14 were average and results given in mM for each SCFA (**b**) or expressed as total concentrations (c). Significant differences based on Wilcoxon test are presented as ∗p<0.05, ∗∗p<0.01, ∗∗∗p<0.001 and ∗∗∗∗p<0.0001. ns: non-significant difference (p>0.05). HD: healthy diet, INU: inulin, LAM: laminarin, RFO: raffinose family oligosaccharides, WD: western diet.

After inoculation of the M-ARCOL model, applying nutritional and physicochemical parameters of the human colonic ecosystem led a shift from faecal to colonic profiles, as described in previous studies (Deschamps et al., 2020; Etienne-Mesmin et al., 2022; Liu et al., 2018; Van den Abbeele et al., 2010). Stabilisation of bacterial composition occurred within 5–7 days of fermentation (data not shown). RDA of bacterial β-diversity confirmed the lack of time effect from day 8 (data not shown). We also observed in the M-ARCOL a clear distinction between lumen and mucus-associated microbiota (Fig. S1). The mucosal compartment exhibited a higher number of taxa, as shown by α-diversity indexes (Fig. 3a and b) but was less colonised than the luminal phase (total bacteria reaching 6–7 Log_10_ 16S rDNA copies/g *versus* 9 Log_10_ 16S rDNA copies/g, data not shown). The mucosal compartment was characterised by an enrichment in *Bacillota* (formerly *Firmicutes*)*,* especially from *Lachnospiraceae* and *Acidaminococcaceae* families, and allowed the preservation of rare taxa such as *Sporanaerobacter* or *Succiniclasticum* genus for example (Figs. 4 and S2). On the contrary, the luminal compartment was enriched in *Bacteroidota* (especially *Prevotellaceae*) and in *Ruminococcus*_E from *Bacillota* (Figs. 4 and S2). Of interest, the inter-individual variabilities observed between the three donors in stool samples was maintained in the *in vitro* colon model, as shown by PCoA analysis (p<0.05) (Fig. S1) and gut microbiota profiles at the family and genus levels (Fig. 4). As for bacterial composition, stabilisation of bacterial activities occurred within 5–7 days of fermentation (data not shown). For all bioreactors, gas ratios were less donor dependent than bacterial composition and were stabilised with a majority of CO_2_ (around 70 %) for each donor and each condition (Fig. 5a). Only a small amount (<2 %) of O_2_ was detected, indicating very good anaerobic conditions. Like for gases, SCFA profiles were less donor dependent than bacterial composition (Fig. 6a). In most cases, acetate was the main metabolite produced (between 50 and 60 %) followed by propionate (around 25 %) and butyrate (between 15 and 25 %). Consequently, further results of bacterial composition and activities were focused on the stabilised period, *i.e.*, from day 8 to day 14.

### 3.2 Effect of western diet on microbiota composition and metabolic activities

**Fig. 7:**
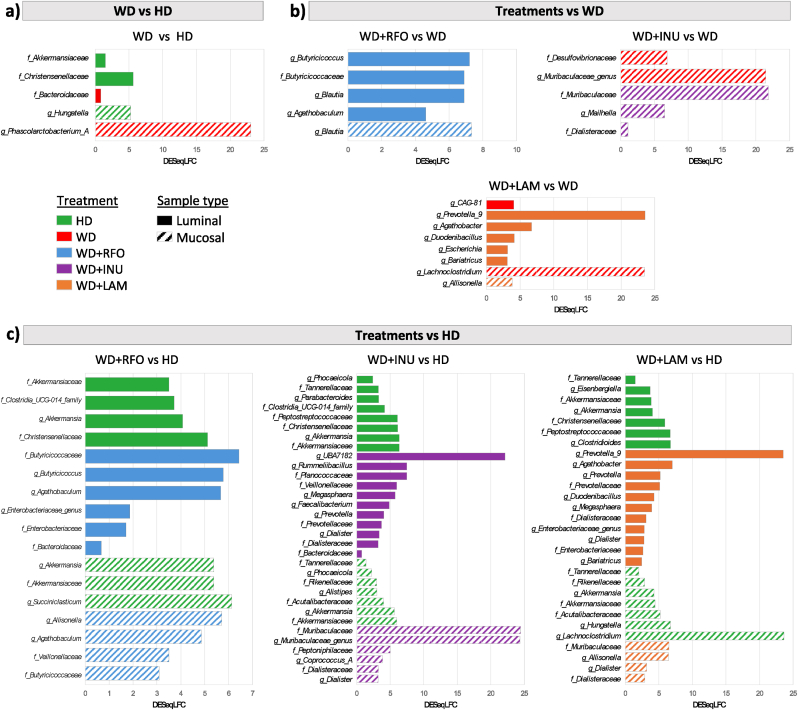
Differential analysis of the impact of western diet and prebiotic supplementation on lumen microbiota and mucosal composition Bacterial microbiota composition was analysed by 16S rRNA Metabarcoding. Differential analyses were further performed on even days 8–14 (with the results from all three donors pooled) to highlight differentially expressed family and genus between the five conditions, using healthy or western diet as control conditions. Differential analyses based on DESeq2, metagenomeSeq and metacoder (p<0.05) methods were performed to between healthy and western diet (a), between prebiotic supplementation and non-supplemented western diet (b) and between prebiotic supplementation under western diet and healthy diet (c). The solid histogram corresponds to luminal samples and hatched histogram corresponds to mucosal samples. Green: HD, red: WD, blue: WD + RFO, purple: WD + INU, orange: WD + LAM. HD: healthy diet, INU: inulin, LAM: laminarin, RFO: raffinose family oligosaccharides, WD: western diet.

Applying WD conditions in the M-ARCOL led to a significantly lower α-diversity (observed ASVs and Shannon indexes) in both the luminal (medians values for WD: 73 *versus* HD: 85.5, p<0.05; WD: 2.3 *versus* HD: 2.7, p<0.01, respectively) and mucosal (medians values for WD: 74.5 *versus* HD: 100, p<0.01; WD: 2.8 *versus* HD: 3.5, p<0.05, respectively) compartments compared to healthy diet (Fig. 3a and b). Additionally, RDA analysis of bacterial β-diversity showed distinct clustering between HD and WD samples in luminal or mucosal compartments (Fig. 3c), more marked for Donor_2 (Fig. 3d). Bacterial profiles at the phylum (Fig. S2), family (Fig. 4a) and genus (Fig. 4b) levels also clearly indicated that the impact of WD on gut microbiota was donor-dependant (e.g., enrichment in *Prevotellaceae* in Donor_2 with WD). When all donors were pooled, differential analysis mostly revealed that *Akkermansiaceae and Christensenellaceae* were significantly depleted in the luminal phase under WD, while *Bacteroidaceae or Phascolarctobacterium_A* were more abundant (Fig. 7a). Regarding gut microbiota activity, the impact of WD was also donor-dependent, but to a lesser extent than on bacterial composition (Fig. 5, Fig. 6a). Compared to HD, WD led to significantly higher CO_2_ (medians: 73.8 % *versus* 63.8 %) and lower H_2_ (medians: 0.4 % *versus* 1.7 %) percentages (Fig. 5b). The gut microbiota also tended to produce more gas under WD than HD (Fig. 5c). SCFA production was not impacted by WD, except a significant (p<0.05) higher propionate concentration in WD (22.2 mM) compared to HD (13.5 mM) (Fig. 6b and c).

### 3.3 Effects of various prebiotics on bacterial diversity

In the luminal compartment of the M-ARCOL (Fig. 3a), addition of the different prebiotics (RFO, INU and LAM) did not impact the number of observed ASVs and Shannon's index compared to the non-supplemented WD. When compared with HD, prebiotics treatments failed to restore α-diversity (p<0.05), except for WD + RFO which showed no significant difference with HD, but for the Shannon index only. When the different prebiotics were compared (under WD condition), WD + INU was associated with the lower Shannon index (median: 1.9), while WD + RFO exhibited the highest one (median: 2.5). Similar trends were observed in the mucosal compartment, with no significant variation of observed ASVs and Shannon index under prebiotic treatment compared to non-supplemented WD, and all prebiotics did not restore α-diversity level close to the HD control, except RFO for the Shannon index (Fig. 3b). RDA of bacterial β-diversity (removing donor effect) showed a clear clustering depending on prebiotic treatments in both the luminal and mucosal compartments (Fig. 3c). When analysed individually, all donors showed different responses to prebiotic treatments (Figs. 3d and S1). For example, in the luminal phase, all prebiotic treatments cannot be separated from HD and WD samples for Donor_3, while clear clustering was observed between HD, WD and WD + RFO for Donor_2 (Fig. 3d). In the mucosal phase, WD + RFO and WD + INU were clearly clustering differentially for Donor_1, while it was rather WD + RFO and WD + LAM for Donor_2 (Fig. 3d).

### 3.4 Effects of prebiotics on bacterial composition

During fermentation, total bacteria were quantified, showing a constant level of around 10^9^ copies 16S rDNA/g for luminal samples and between 10^6^ and 10^7^ copies 16S rDNA/g for mucosal samples, with no impact of prebiotic supplementation (data not shown). The analysis of bacterial populations at the phylum (Fig. S2), family (Fig. 4a) and genus (Fig. 4b) levels showed clearly donor-dependent effects of prebiotic treatments. For example, Donor_1 showed an important shift in bacterial composition compared to non-supplemented WD with all prebiotic treatments, with an enrichment in *Ruminococcaceae*, especially *Ruminococcus_E* allowing to restore this population found under HD (Fig. 4). The same donor was the most impacted by the WD + LAM treatment, with the highest enrichment of *Acidaminococcus* and *Prevotella* in both micro-environments and *Veillonellaceae* family and *Dialister* genus in the mucosal compartment only. In comparison, Donor_3 was almost not impacted by prebiotic treatments compared to HD and WD controls (Fig. 4). When all donors were pooled, differential analysis revealed that only *Butyricicoccaceae* family and more specifically its genus *Agathobaculum* and *Butyricicoccus* were significantly enriched with WD + RFO in the luminal samples compared to WD (Fig. 7b). In addition, *Blautia* genus was more abundant when adding this prebiotic in both luminal and mucosal compartments. For WD + LAM, differential analysis confirmed that *Prevotella_9* and *Agathobacter* genera were significantly more abundant in luminal compared to non-supplemented WD (Fig. 7b). At both family and genus levels, WD + INU was associated with no shift in bacterial composition in luminal samples. On the contrary, in the mucosal phase, WD + INU led to a significant increase in *Dialisteraceae* and *Muribaculaceae* while *Desulfovibrionaceae* was depleted (Fig. 7b). Compared to HD, *Akkermansiaceae* and *Christensenellaceae* were less abundant in luminal whereas *Akkermansiaceae* and *Rikenellaceae* were less abundant in mucosal compartment in prebiotics treatments (Fig. 7c). Comparing WD + RFO and HD, *Butyricicoccaceae* genus, *Butyricicoccus* and *Agathobaculum*, were more abundant with the prebiotic (Fig. 7c). When differential analyses were performed to compare prebiotics treatments under WD and HD (Fig. 7c), a greater number of bacterial populations were impacted with WD + INU and WD + LAM compared to WD + RFO. WD + RFO was mainly associated with a significant bloom in *Butyricicoccaceae* (*Butyricicoccus*) in both luminal and mucosal microenvironments. WD + INU was associated with an enrichment on the luminal samples in *Prevotellaceae* and *Veillonellaceae* families and *Prevotella* and *Faecalibacterium* genera, while *Muribaculaceae* was the most positively affected in the mucosal samples. WD + LAM showed an enrichment in *Prevotellaceae* and *Enterobacteriaceae* families and *Agathobacter, Megasphaera, Prevotella* and *Prevotella_9* genera in the luminal compartment (Fig. 7c). Of note, both WD + INU and WD + LAM, but not WD + RFO led to a significant increase in *Dialisteraceae* (*Dialister*) in both microenvironments. When prebiotic treatments were compared one with another (Fig. S3), differential analyses revealed that *Butyricicoccus* and *Blautia* genera were more abundant with WD + RFO, while *Faecalibacterium* was enriched with WD + INU and *Agathobacter* and *Prevotella* genera with WD + LAM.

### 3.5 Effects of prebiotics on microbiota metabolic activities

In addition to microbial diversity and composition, the effects of the three tested prebiotics were assessed by monitoring microbial activities, through atmospheric gases (Fig. 5) and SCFA measurements (Fig. 6), with, for both, donor-dependent effect of supplementations (Fig. 5, Fig. 6a). In all donors, supplementation of WD with prebiotics had no impact on O_2_ and CO_2_ percentages compared to the non-supplemented WD. On the contrary, prebiotics led to a significant (p<0.001) increase in H_2_ production, with a median value of 4.6 % with WD + RFO and 5.3 % with WD + INU *versus* 0.4 % with the control WD (Fig. 5b). When compared to non-supplemented WD, the total gas volume tended to increase with all prebiotic treatments (Fig. 5c), but these differences only reached significance with WD + INU (150 mL *versus* 80.5 mL, p<0.05). When comparisons were made with HD control, the major effect of all prebiotic treatments was a significant increase in CO_2_ percentages (medians around 73 % *versus* 64 %), and an increase in total gas production, significant for WD + INU (p<0.01) and WD + LAM (p<0.05). When prebiotic treatments were compared under WD condition, the most striking effect was a significant (p<0.05) higher H_2_ production with WD + RFO (4.6 %) and WD + INU (5.3 %) compared to WD + LAM (1.3 %) (Fig. 5b).

Regarding SCFA, acetate was the main metabolite produced, except for WD + INU in Donor_1 and 2 (Fig. 6a). Probiotic supplementation led to no significant change in main SCFA concentrations compare to non-supplemented WD (Fig. 6b), except for WD + RFO which induced a significant increase in butyrate concentrations (*i.e.*, 16.4 mM–12 mM, p<0.001). When compared to HD control, WD + LAM was associated with a significant higher propionate concentration (p<0.05), while WD + RFO led to an increase in propionate (p<0.05) and butyrate (p<0.001). In addition, supplementation with all prebiotics did not result in any change in total SCFA concentrations when compared to both WD and HD controls (Fig. 6c). When prebiotic treatments were compared to each other under WD condition (Fig. 6b), WD + RFO results in significant higher concentrations of acetate than WD + INU (38.3 mM *versus* 24.3 mM, p<0.0001) and WD + LAM (38.3 mM *versus* 25.9 mM, p<0.01) and was associated to greater butyrate levels than WD + LAM (16.4 mM *versus* 10.5 mM, p<0.001). Of note, WD + RFO tended to present higher concentrations of total SCFA than the other two prebiotics, resulting in a significant increase in total SCFA (75.1 mM *versus* 60.2 mM, p<0.01) with WD + RFO compared to WD + LAM (Fig. 6c).

### 3.6 Correlation between microbiota composition and function depending on prebiotic treatment

**Fig. 8:**
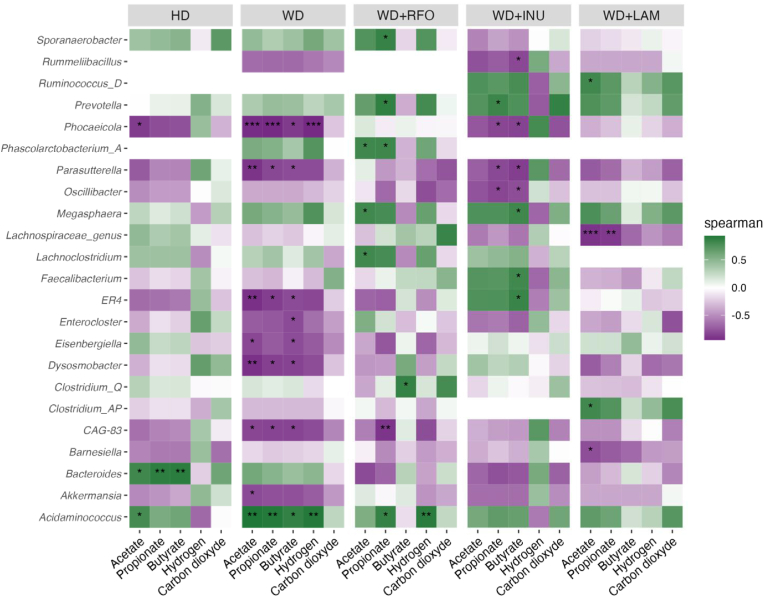
Spearman correlation between microbiota composition at the genus level and gas or SCFA production. Spearman correlation between microbiota composition at the genus level and gas or SCFAs production are represented by a heatmap depending on tested conditions (HD, WD, WD + INU, WD + LAM and WD + RFO). Green color indicates a positive correlation while purple denotes a negative correlation. Color shading gives the magnitude of the association. Asterisks indicate p-value significance: ∗p<0.05, ∗∗p<0.01 and ∗∗∗p<0.001. HD: healthy diet, INU: inulin, LAM: laminarin, RFO: raffinose family oligosaccharides, WD: western diet.

To further reveal the correlations between relative abundances of bacterial genera and gas or SCFA production, Spearman correlation analyses were performed (Fig. 8). Clearly different heatmaps were obtained between HD and WD, and when the different prebiotics were added. Whatever the tested conditions, no significant correlation was evidenced between CO_2_ and any bacterial populations. On the contrary, the highest number of significant correlations was obtained with butyrate. Under HD control, the main correlation was the positive association of *Bacteroides* with the three main SCFAs. When analysing WD control, most of correlations was negative ones, with *Phocaeicola* inversely correlated with SCFA and H_2_ (p<0.05) and *Akkermansia* with acetate (p<0.05). *Acidaminococcus* was the only one to be positively associated with the three main SCFA and H_2_ (p<0.05). Regarding WD + RFO, *Acidaminococcus* was positively correlated with propionate and H_2_ (p<0.05). *Megasphaera*, *Lachnoclostridium*, *Prevotella* and *Clostridium_Q* were also positively associated with at least one of the SCFA analysed (p<0.05) and *CAG-83* was the only one to negatively correlate with propionate (p<0.01). Under WD + INU condition, as previously observed for WD + RFO, *Prevotella* was positively correlated with propionate (p<0.05). *Megasphaera*, *Faecalibacterium* and *ER4* showed a significant positive correlation with butyrate, while a negative correlation was observed between *Phocaeicola*, *Parasutterella* and *Oscillibacter* and propionate or butyrate production (p<0.05). No significant correlation was highlighted for acetate and H_2_ with WD + INU, whatever the bacterial population. Lastly, regarding WD + LAM, few correlations were evidenced, and none for butyrate and H_2_. However, a positive correlation was found between *Ruminococcus_D* or *Clostridium_AP* and acetate, while *Barnesiella* and *Lachnospiraceae_genus* were negatively correlated to this SCFA. *Lachnospiraceae_genus* was also negatively correlated with propionate.

## 4 Discussion

Characterised by a high content in fat and sugar and a low fibre input, WD has shown numerous deleterious consequences on human health, such as gut inflammation, increased intestinal permeability and association with highly prevalent pathologies like diabetes, heart diseases or obesity (Clemente-Suárez et al., 2023). Most of the time, WD is part of the obesity disease, which is associated with gut microbiota dysbiosis, defined by a low bacterial diversity, a drop in health-related populations such as *Akkermansiaceae*, *Rikenellaceae*, *Christensenellaceae* and *Ruminococcaceae,* and an increase in proinflammatory bacteria, such as Proteobacteria (Clemente-Suárez et al., 2023; Malesza et al., 2021; Crovesy et al., 2020). Most of those changes were accurately reproduced in our *in vitro* gut M-ARCOL model fed with a western-like diet, already described in own of our previous study (O'Sullivan et al., 2024). In particular, in line with *in vivo* data, under WD condition in the *in vitro* gut model, bacterial diversity was reduced and both health-related populations *Akkermansiaceae* and *Christensenellaceae* families were depleted compared to the HD control (O'Sullivan et al., 2024; Yassour et al., 2016; Peters et al., 2018; Jinatham et al., 2018; de la Cuesta-Zuluaga et al., 2018; Goodrich et al., 2016; Ettehad Marvasti et al., 2020; Dao et al., 2019). Of note, at the species level, *Akkermansia muciniphila* is well-known for its anti-obesity properties in humans (Cani, 2018; Schneeberger et al., 2015) and *Christensenella minuta* protects from diet-induced obesity in rodents and was inversely correlated to BMI in humans (Mazier et al., 2021; Waters and Ley, 2019). Regarding metabolic activities, we noted no significant change in SCFA and gas production, except increases in CO_2_ and propionate. To the best of our knowledge, there is no *in vivo* data about gas composition in the human colon under WD or obese conditions. In obese patients, total fecal SCFA concentrations are higher compared to healthy volunteers, but with similar proportion of the main SCFA (Schwiertz et al., 2010; Rahat-Rozenbloom et al., 2014; Fernandes et al., 2014; Fernández-Navarro et al., 2017; Dugas et al., 2018). In our study, the increase in propionate concentrations may be (at least partly) associated with a rise in *Phascolarctobacterium_A*, an acetate/propionate producer (Wu et al., 2017), also highlighted *in vivo* in obese children (Chen et al., 2020).

To counteract the effects of WD on human gut microbiota and prevent associated disease like obesity, prebiotics appears as a relevant strategy (Martinez et al., 2017; Paone et al., 2022). In this context, it is necessary to identify new prebiotics from various sources, other than milk or cereals (Bamigbade et al., 2022; Shannon et al., 2021) with acknowledged health effects and enhance our knowledges on their mechanisms of action in relation with human gut microbiota modulation. In particular, RFO and LAM have already demonstrated their anti-obesity, anti-cancer, anti-inflammatory or anti-allergic properties (Dai et al., 2019; Moghaddam et al., 2018; Muthukumaran et al., 2018; Nagura et al., 2002; Shakappa et al., 2018; Watanabe et al., 2004; Nguyen et al., 2016; Song et al., 2017; Zhang et al., 2023), but it is necessary to delve deeper into their interactions with human gut microbiota before making definite conclusions about their prebiotic potential. With this objective in mind, our “dysbiotic” *in vitro* model was used in the present study to evaluate the impact of RFO from chickpeas and LAM from algae, in comparison to the well-known INU from agave, on human colonic microbiota composition and metabolic activities. Our ultimate goal was to determine their ability to restore microbiota balance, in comparison with two different controls, a non-supplemented WD condition and a HD control.

Our results revealed that, whatever the tested compound, prebiotic supplementation under WD condition was not able to restore a “healthy-like” microbiota, in terms of bacterial diversity and composition. The lack of RFO and LAM effect on bacterial diversity is in line with the results observed in previous *in vivo* studies (Fernando et al., 2010; Nguyen et al., 2016; Zhang et al., 2023). Nevertheless, addition of prebiotics induced an increase in some beneficial bacteria in the M-ARCOL compared to the non-supplemented condition. Indeed, treatment with RFO led to a raise in *Butyricicoccaceae* (*Butyricicoccus* and *Agathobaculum*) family and *Blautia* genus. All these bacteria have already shown interesting probiotic effects in previous studies (Eeckhaut et al., 2013; Go et al., 2024; Lee et al., 2022; Liu et al., 2021b; Wang et al., 2021). In addition, *Butyricicoccaceae* are well-known producers of butyrate with recognized anti-inflammatory effects (Ahn et al., 2016; Eeckhaut et al., 2008; Takada et al., 2016). Butyrate has also yet exhibited health benefits in the context of obesity, diabetes, neurotoxicity or atherosclerosis induced by high fat diet in mice or in human (Bayazid et al., 2022; Coppola et al., 2021; Du et al., 2020; Mayorga-Ramos et al., 2022). In line with those data, RFO was the only prebiotic associated with a significant increase in butyrate concentrations in our M-ARCOL fed with WD. RFO supplementation was also related to a clear peak in H_2_ production. This may be linked to *Butyricicoccus* genus known to produce H_2_ (Eeckhaut et al., 2008) or *Acidaminococcus* (Russell, 2015), which was positively correlated with H_2_ production in our *in vitro* study (Spearman correlations). Several previous studies have investigated RFO effects *in vitro* (human faecal batch culture) or *in vivo* (animals or humans) and revealed an increase in *Bifidobacterium* and *Lactobacillus* together with a decrease in *Clostridium* (Amorim et al., 2020; Dai et al., 2019; Fernando et al., 2010; Bai et al., 2018; Kanwal et al., 2023). On the contrary, *Bifidobacterium* and *Lactobacillus* were not detected in the M-ARCOL and *Clostridiaceae* were not impacted by RFO treatment. Even if our study confirmed that RFO exhibit interesting prebiotic effects, the main concern associated with their use in humans seems to be their ability to induce excessive gas production (Elango et al., 2022; Dahl et al., 2014). This has been confirmed in the present study by a 2-fold higher gas production with RFO compared to the two controls (even if not significant), in accordance with results obtained in human *in vitro* batch culture (Amorim et al., 2020). It is therefore important to determine the appropriate dose that allows RFO to reveal their prebiotic potential but without excessive flatulence.

Regarding LAM, supplementation with this prebiotic led to an increase in *Prevotella* family and genera, but also a raise in other families, such as *Agathobacter*, *Dialister* and *Megasphaera*. Very few studies have yet investigated the effects of LAM alone on human gut microbiota, highlighting the novelty of our results. Most of available studies have been performed on brown seaweed extracts containing laminarin, but also other polysaccharides, limiting their interpretation (Shannon et al., 2021; Charoensiddhi et al., 2016; Liu et al., 2023; Lynch et al., 2010; Strain et al., 2020). Among the available data on LAM and gut microbiota from human origin, the first revealed an increase in *Bifidobacterium* and *Bacteroides* with LAM treatment (Seong et al., 2019), while the second showed no difference in *Bacteroides*, *Biﬁdobacterium* and *Lactobacillus* abundances (Devillé et al., 2007). In this study, *Bifidobacterium* and *Lactobacillus* were not detected in the bioreactors and LAM had a donor dependent impact on *Bacteroides*. However, the increase in *Prevotellaceae* observed in the M-ARCOL with LAM was also observed in the caecum of pigs fed with laminarin rich extract (Vigors et al., 2020). Of interest, a study revealed that LAM supplementation in pigs led to an increase in *muc2* and *muc4* expression, which are involved in mucin synthesis (Smith et al., 2011). Since WD was associated with a decrease in *muc2* expression in mice (Stolfi et al., 2023), these potential properties of LAM would deserve more attention. Regarding gut microbial activities, no significant effect on gas and SCFA was highlighted in our *in vitro* M-ARCOL model after LAM treatment. On the contrary, previous *in vitro* studies with human faecal cultures revealed an increase in main and total SCFA production (Seong et al., 2019; Devillé et al., 2007), but, to the best of our knowledge, no study has yet investigated LAM impact on gas production. Those results raised the question of LAM digestibility by occidental gut microbiome since algae are not frequent in this diet, in comparison to Asian food. However, recent studies have demonstrated that some *Bacteroides* species, present in Asian and Western microbiota, could metabolize LAM by producing laminarinase and β-glucosidase (Strain et al., 2020; Charoensiddhi et al., 2020) and in the study from Devillé et al., more than 90 % of LAM were degraded in less than 24 h incubation (Devillé et al., 2007). When compared, those results suggest that LAM degradation and fermentation might be donor- or dose-dependent.

When all donors were pooled, we found that RFO and LAM did not exhibit more marked effects on metabolic activities (as followed by SCFA and gas production) than INU. Regarding the impact on microbiota composition, the three prebiotics promoted distinct populations, mainly *Blautia* and *Butyricicoccaceae* for RFO, *Faecalibacterium* for INU or *Agathobacter* for LAM, suggesting different mechanisms of actions mediated by gut microbes. However, the interpretation of those data must consider that, even if a dose of 2.5 g/day was administered for all three vegetal extracts, the prebiotics were not pure, with potential dose-dependent effects of the active compounds, but also of other components such as proteins or minerals. In addition, it should be highlighted that the most promising effects *in vitro* were observed with RFO, which is the least concentrated compound in extracts. Of note also, recent studies on mice with diet-related obesity have shown that prolonged consumption of soluble fibre such as inulin can lead to hepatocellular carcinomas (Singh et al., 2018). Those results highlight the necessity to investigate potential harmful effects in human of “prebiotics” and rethink the way to use food supplements towards a personalized custom to promote health in a safer way (Bretin and Chassaing, 2019). Indeed, human gut microbiota composition is largely individual dependent (Eckburg et al., 2005; Jiao et al., 2021) and this inter-individual variability was retained in the M-ARCOL model at all taxonomic levels. In addition, a donor dependent response to prebiotic treatments was observed for both lumen and mucus-associated bacteria in the *in vitro* gut model. This shows that the M-ARCOL model is able to evaluate which prebiotic has the best chance to positively impact gut microbes depending on the individual, opening the possibility to better define responders and non-responders to prebiotic supplementation and move towards personalized nutrition. To further investigate the effect of prebiotics on host responses, as mediated by gut microbiota, M-ARCOL could be combined in next studies with intestinal epithelial cells (Fournier et al., 2023), immune cells or hepatocytes in order to follow immune, inflammation or antioxidant pathways.

## CRediT authorship contribution statement

**Ophélie Uriot:** Conceptualization, Data curation, Formal analysis, Methodology, Investigation, Visualization, Writing – original draft, preparation, Writing – review & editing. **Clémence Defois-Fraysse:** Writing – original draft, preparation, Writing – review & editing. **Ingrid Couturier:** Formal analysis, Investigation, Visualization, Writing – original draft, preparation, Writing – review & editing. **Charlotte Deschamps:** Data curation, Visualization, Writing – original draft, preparation, Writing – review & editing. **Claude Durif:** Investigation, Formal analysis, Writing – review & editing. **Cyril Chaudemanche:** Project administration, Funding acquisition, Writing – review & editing. **Assia Dreux-Zigha:** Project administration, Funding acquisition, Writing – review & editing. **Stéphanie Blanquet-Diot:** Conceptualization, Funding acquisition, Resources, Project administration, Supervision, Validation, Writing – original draft, preparation, Writing – review & editing.

## Data availability

Raw sequence data are available at NCBI under the Sequence Read Archive database in the BioProject n◦PRJNA1168894. Other data will be made available on request.

## Ethics statement

This study was a noninterventional study with no addition to usual clinical care. Appropriate measures were taken to protecting the rights and privacy of all participants. The donors provided consent for the analysis and publication of the findings for faecal samples in the specific context of this study. The work described has been carried out in accordance with The Code of Ethics of the World Medical Association (Declaration of Helsinki) for experiments involving humans.

## Fundings sources

This study was performed in the frame of the PSPC RESTORBIOME (2019–2023). This project was supported by the Program Investissement Avenir operated by BpiFrance, 10.13039/100004757General Mills/Yoplait and Greencell industrial partners, Clermont Auvergne Metropole and Région Auvergne Rhône Alpes.

## Declaration of competing interest

The authors declare the following financial interests/personal relationships which may be considered as potential competing interests: Stephanie Blanquet-Diot reports financial support was provided by BPI France. Stephanie Blanquet-Diot reports financial support was provided by Clermont Auvergne Metropole. Stephanie Blanquet-Diot reports financial support was provided by Auvergne-Rhone-Alpes Region. Clemence Defois reports a relationship with Greencell that includes: employment. Assia Dreux-Zigha reports a relationship with Greencell that includes: employment. General Mills reports a relationship with Cyril Chaudemanche that includes: employment. The other authors declare that they have no known competing financial interests or personal relationships that could have appeared to influence the work reported in this paper. If there are other authors, they declare that they have no known competing financial interests or personal relationships that could have appeared to influence the work reported in this paper.

## Data Availability

Data will be made available on request.

## References

[bib1] Ahn S., Jin T.-E., Chang D.-H., Rhee M.-S., Kim H.J., Lee S.J., Park D.-S., Kim B.-C. (2016). Agathobaculum butyriciproducens gen. nov. sp. nov., a strict anaerobic, butyrate-producing gut bacterium isolated from human faeces and reclassification of Eubacterium desmolans as Agathobaculum desmolans comb. nov. Int. J. Syst. Evol. Microbiol..

[bib2] Amorim C., Silvério S.C., Cardoso B.B., Alves J.I., Pereira M.A., Rodrigues L.R. (2020). *In vitro* fermentation of raffinose to unravel its potential as prebiotic ingredient. Lebensm. Wiss. Technol..

[bib3] Bai G., Tsuruta T., Nishino N. (2018). Dietary soy, meat, and fish proteins modulate the effects of prebiotic raffinose on composition and fermentation of gut microbiota in rats. Int. J. Food Sci. Nutr..

[bib4] Bamigbade G.B., Subhash A.J., Kamal-Eldin A., Nyström L., Ayyash M. (2022). An updated review on prebiotics: insights on potentials of food seeds waste as source of potential prebiotics. Molecules.

[bib5] Bayazid A.B., Kim J.G., Azam S., Jeong S.A., Kim D.H., Park C.W., Lim B.O. (2022). Sodium butyrate ameliorates neurotoxicity and exerts anti-inflammatory effects in high fat diet-fed mice. Food Chem. Toxicol. Int. J. Publ. Br. Ind. Biol. Res. Assoc..

[bib6] Bretin A., Chassaing B. (2019). [Inulin: the beauty and the beast]. Med. Sci. MS.

[bib7] Callahan B.J., McMurdie P.J., Rosen M.J., Han A.W., Johnson A.J.A., Holmes S.P. (2016). DADA2: high-resolution sample inference from Illumina amplicon data. Nat. Methods.

[bib8] Cani P.D. (2018). Human gut microbiome: hopes, threats and promises. Gut.

[bib9] Capone S.H., Dufresne M., Rechel M., Fleury M.-J., Salsac A.-V., Paullier P., Daujat-Chavanieu M., Legallais C. (2013). Impact of alginate composition: from bead mechanical properties to encapsulated HepG2/C3A cell activities for in vivo implantation. PLoS One.

[bib10] Cardoso B.B., Amorim C., Silvério S.C., Rodrigues L.R. (2021). Novel and emerging prebiotics: advances and opportunities. Adv. Food Nutr. Res..

[bib11] Charoensiddhi S., Conlon M.A., Vuaran M.S., Franco C.M.M., Zhang W. (2016). Impact of extraction processes on prebiotic potential of the brown seaweed *Ecklonia radiata* by *in vitro* human gut bacteria fermentation. J. Funct.Foods.

[bib12] Charoensiddhi S., Abraham R., Su P., Zhang W. (2020). Seaweed and seaweed-derived metabolites as prebiotics. Adv. Food Nutr. Res..

[bib13] Chen X., Sun H., Jiang F., Shen Y., Li X., Hu X., Shen X., Wei P. (2020). Alteration of the gut microbiota associated with childhood obesity by 16S rRNA gene sequencing. PeerJ.

[bib14] Cherry P., Yadav S., Strain C.R., Allsopp P.J., McSorley E.M., Ross R.P., Stanton C. (2019). Prebiotics from seaweeds: an ocean of opportunity?. Mar. Drugs.

[bib15] Clemente-Suárez V.J., Beltrán-Velasco A.I., Redondo-Flórez L., Martín-Rodríguez A., Tornero-Aguilera J.F. (2023). Global impacts of western diet and its effects on metabolism and health: a narrative review. Nutrients.

[bib16] Coppola S., Avagliano C., Calignano A., Berni Canani R. (2021). The protective role of butyrate against obesity and obesity-related diseases. Mol. Basel Switz..

[bib17] Crovesy L., Masterson D., Rosado E.L. (2020). Profile of the gut microbiota of adults with obesity: a systematic review. Eur. J. Clin. Nutr..

[bib18] Dahl W.J., Hanifi A., Zello G.A., Tyler R.T. (2014). Gastrointestinal tolerance to daily canned chickpea intake, can. J. Diet. Pract. Res. Publ. Dietit. Can. Rev. Can. Prat. Rech. En diet. Une publ. Diet. Can..

[bib19] Dai Z., Feng S., Liu A.B., Wang H., Zeng X., Yang C.S. (2019). Protective effects of α-galacto-oligosaccharides against a high-fat/western-style diet-induced metabolic abnormalities in mice. Food Funct..

[bib20] Dao M.C., Belda E., Prifti E., Everard A., Kayser B.D., Bouillot J.-L., Chevallier J.-M., Pons N., Le Chatelier E., Ehrlich S.D., Doré J., Aron-Wisnewsky J., Zucker J.-D., Cani P.D., Clément K. (2019). Akkermansia muciniphila abundance is lower in severe obesity, but its increased level after bariatric surgery is not associated with metabolic health improvement. Am. J. Physiol. Endocrinol. Metab..

[bib21] de la Cuesta-Zuluaga J., Corrales-Agudelo V., Carmona J.A., Abad J.M., Escobar J.S. (2018). Body size phenotypes comprehensively assess cardiometabolic risk and refine the association between obesity and gut microbiota. Int. J. Obes..

[bib22] Deschamps C., Fournier E., Uriot O., Lajoie F., Verdier C., Comtet-Marre S., Thomas M., Kapel N., Cherbuy C., Alric M., Almeida M., Etienne-Mesmin L., Blanquet-Diot S. (2020). Comparative methods for fecal sample storage to preserve gut microbial structure and function in an in vitro model of the human colon. Appl. Microbiol. Biotechnol..

[bib23] Devillé C., Gharbi M., Dandrifosse G., Peulen O. (2007). Study on the effects of laminarin, a polysaccharide from seaweed, on gut characteristics. J. Sci. Food Agric..

[bib24] Du Y., Li X., Su C., Xi M., Zhang X., Jiang Z., Wang L., Hong B. (2020). Butyrate protects against high-fat diet-induced atherosclerosis via up-regulating ABCA1 expression in apolipoprotein E-deficiency mice. Br. J. Pharmacol..

[bib25] Dugas L.R., Bernabé B.P., Priyadarshini M., Fei N., Park S.J., Brown L., Plange-Rhule J., Nelson D., Toh E.C., Gao X., Dong Q., Sun J., Kliethermes S., Gottel N., Luke A., Gilbert J.A., Layden B.T. (2018). Decreased microbial co-occurrence network stability and SCFA receptor level correlates with obesity in African-origin women. Sci. Rep..

[bib26] Eckburg P.B., Bik E.M., Bernstein C.N., Purdom E., Dethlefsen L., Sargent M., Gill S.R., Nelson K.E., Relman D.A. (2005). Diversity of the human intestinal microbial flora. Science.

[bib27] Eeckhaut V., Van Immerseel F., Teirlynck E., Pasmans F., Fievez V., Snauwaert C., Haesebrouck F., Ducatelle R., Louis P., Vandamme P. (2008). Butyricicoccus pullicaecorum gen. nov., sp. nov., an anaerobic, butyrate-producing bacterium isolated from the caecal content of a broiler chicken. Int. J. Syst. Evol. Microbiol..

[bib28] Eeckhaut V., Machiels K., Perrier C., Romero C., Maes S., Flahou B., Steppe M., Haesebrouck F., Sas B., Ducatelle R., Vermeire S., Van Immerseel F. (2013). Butyricicoccus pullicaecorum in inflammatory bowel disease. Gut.

[bib29] Elango D., Rajendran K., Van der Laan L., Sebastiar S., Raigne J., Thaiparambil N.A., El Haddad N., Raja B., Wang W., Ferela A., Chiteri K.O., Thudi M., Varshney R.K., Chopra S., Singh A., Singh A.K. (2022). Raffinose family oligosaccharides: friend or foe for human and plant health?. Front. Plant Sci..

[bib30] Etienne-Mesmin L., Meslier V., Uriot O., Fournier E., Deschamps C., Denis S., David A., Jegou S., Morabito C., Quinquis B., Thirion F., Oñate F.P., Le Chatelier E., Ehrlich S.D., Blanquet-Diot S., Almeida M. (2022). <em>In vitro</em> modelling of oral microbial invasion in the human colon. bioRxiv.

[bib31] Ettehad Marvasti F., Moshiri A., Taghavi M.S., Riazi S., Taati M., Sadati S.F., Ghaheri A., Masoomi M., Vaziri F., Fateh A., Rohani P., Tarashi S., Masotti A., Ahmadi Badi S., Siadat S.D. (2020). The first report of differences in gut microbiota composition between obese and normal weight Iranian subjects, Iran. Biomed. J..

[bib32] Fernandes J., Su W., Rahat-Rozenbloom S., Wolever T.M.S., Comelli E.M. (2014). Adiposity, gut microbiota and faecal short chain fatty acids are linked in adult humans. Nutr. Diabetes.

[bib33] Fernández-Navarro T., Salazar N., Gutiérrez-Díaz I., de Los Reyes-Gavilán C.G., Gueimonde M., González S. (2017). Different intestinal microbial profile in over-weight and obese subjects consuming a diet with low content of fiber and antioxidants. Nutrients.

[bib34] Fernando W.M.U., Hill J.E., Zello G.A., Tyler R.T., Dahl W.J., Van Kessel A.G. (2010). Diets supplemented with chickpea or its main oligosaccharide component raffinose modify faecal microbial composition in healthy adults. Benef. Microbes.

[bib35] Fournier E., Denis S., Dominicis A., Van de Wiele T., Alric M., Mercier-Bonin M., Etienne-Mesmin L., Blanquet-Diot S. (2022). A child is not an adult: development of a new in vitro model of the toddler colon. Appl. Microbiol. Biotechnol..

[bib36] Fournier E., Leveque M., Ruiz P., Ratel J., Durif C., Chalancon S., Amiard F., Edely M., Bezirard V., Gaultier E., Lamas B., Houdeau E., Lagarde F., Engel E., Etienne-Mesmin L., Blanquet-Diot S., Mercier-Bonin M. (2023). Microplastics: what happens in the human digestive tract? First evidences in adults using in vitro gut models. J. Hazard Mater..

[bib37] Gibson G.R., Hutkins R., Sanders M.E., Prescott S.L., Reimer R.A., Salminen S.J., Scott K., Stanton C., Swanson K.S., Cani P.D., Verbeke K., Reid G. (2017). Expert consensus document: the International Scientific Association for Probiotics and Prebiotics (ISAPP) consensus statement on the definition and scope of prebiotics. Nat. Rev. Gastroenterol. Hepatol..

[bib38] Go J., Maeng S.-Y., Chang D.-H., Park H.-Y., Min K.-S., Kim J.-E., Choi Y.-K., Noh J.-R., Ro H., Kim B.-C., Kim K.-S., Lee C.-H. (2024). Agathobaculum butyriciproducens improves ageing-associated cognitive impairment in mice. Life Sci..

[bib39] Goodrich J.K., Davenport E.R., Beaumont M., Jackson M.A., Knight R., Ober C., Spector T.D., Bell J.T., Clark A.G., Ley R.E. (2016). Genetic determinants of the gut microbiome in UK Twins. Cell Host Microbe.

[bib40] Hills R.D., Pontefract B.A., Mishcon H.R., Black C.A., Sutton S.C., Theberge C.R. (2019). Gut microbiome: profound implications for diet and disease. Nutrients.

[bib41] Jiao L., Kourkoumpetis T., Hutchinson D., Ajami N.J., Hoffman K., White D.L., Graham D.Y., Hair C., Shah R., Kanwal F., Jarbrink-Sehgal M., Husain N., Hernaez R., Hou J., Cole R., Velez M., Ketwaroo G., Kramer J., El-Serag H.B., Petrosino J.F. (2021). Spatial characteristics of colonic mucosa-associated gut microbiota in humans. Microb. Ecol..

[bib42] Jinatham V., Kullawong N., Kespechara K., Gentekaki E., Popluechai S. (2018). Comparison of gut microbiota between lean and obese adult Thai individuals. Microbiol. Biotechnol. Lett..

[bib43] Kanwal F., Ren D., Kanwal W., Ding M., Su J., Shang X. (2023). The potential role of nondigestible Raffinose family oligosaccharides as prebiotics. Glycobiology.

[bib44] Klindworth A., Pruesse E., Schweer T., Peplies J., Quast C., Horn M., Glöckner F.O. (2013). Evaluation of general 16S ribosomal RNA gene PCR primers for classical and next-generation sequencing-based diversity studies. Nucleic Acids Res..

[bib45] Lambrecht E., Van Coillie E., Boon N., Heyndrickx M., Van de Wiele T. (2021). Transfer of antibiotic resistance plasmid from commensal E. coli towards human intestinal microbiota in the M-SHIME: effect of E. coli dosis, human individual and antibiotic use. Life.

[bib46] Lee D.W., Ryu Y.-K., Chang D.-H., Park H.-Y., Go J., Maeng S.-Y., Hwang D.Y., Kim B.-C., Lee C.-H., Kim K.-S. (2022). Agathobaculum butyriciproducens shows neuroprotective effects in a 6-OHDA-induced mouse model of Parkinson's disease. J. Microbiol. Biotechnol..

[bib47] Liu L., Firrman J., Tanes C., Bittinger K., Thomas-Gahring A., Wu G.D., Van den Abbeele P., Tomasula P.M. (2018). Establishing a mucosal gut microbial community in vitro using an artificial simulator. PLoS One.

[bib48] Liu C., Cui Y., Li X., Yao M. (2021). microeco: an R package for data mining in microbial community ecology. FEMS Microbiol. Ecol..

[bib49] Liu X., Mao B., Gu J., Wu J., Cui S., Wang G., Zhao J., Zhang H., Chen W. (2021). Blautia-a new functional genus with potential probiotic properties?. Gut Microb..

[bib50] Liu T., Zhang M., Asif Im, Wu Y., Li B., Wang L. (2023). The regulatory effects of fucoidan and laminarin on functional dyspepsia mice induced by loperamide. Food Funct..

[bib51] Lynch M.B., Sweeney T., Callan J.J., O'Sullivan J.T., O'Doherty J.V. (2010). The effect of dietary Laminaria-derived laminarin and fucoidan on nutrient digestibility, nitrogen utilisation, intestinal microflora and volatile fatty acid concentration in pigs. J. Sci. Food Agric..

[bib52] Malesza I.J., Malesza M., Walkowiak J., Mussin N., Walkowiak D., Aringazina R., Bartkowiak-Wieczorek J., Mądry E. (2021). High-fat, western-style diet, systemic inflammation, and gut microbiota: a narrative review. Cells.

[bib53] Martin A.M., Sun E.W., Rogers G.B., Keating D.J. (2019). The influence of the gut microbiome on host metabolism through the regulation of gut hormone release. Front. Physiol..

[bib54] Martinez K.B., Leone V., Chang E.B. (2017). Western diets, gut dysbiosis, and metabolic diseases: are they linked?. Gut Microb..

[bib55] Marzorati M., Van den Abbeele P., Bubeck S.S., Bayne T., Krishnan K., Young A., Mehta D., DeSouza A. (2020). Bacillus subtilis HU58 and Bacillus coagulans SC208 probiotics reduced the effects of antibiotic-induced gut microbiome dysbiosis in an M-SHIME® model. Microorganisms.

[bib56] Mayorga-Ramos A., Barba-Ostria C., Simancas-Racines D., Guamán L.P. (2022). Protective role of butyrate in obesity and diabetes: new insights. Front. Nutr..

[bib57] Mazier W., Le Corf K., Martinez C., Tudela H., Kissi D., Kropp C., Coubard C., Soto M., Elustondo F., Rawadi G., Claus S.P. (2021). A new Strain of Christensenella minuta as a potential biotherapy for obesity and associated metabolic diseases. Cells.

[bib58] Moghaddam S.M., Brick M.A., Echeverria D., Thompson H.J., Brick L.A., Lee R., Mamidi S., McClean P.E. (2018). Genetic architecture of dietary fiber and oligosaccharide content in a middle American panel of edible dry bean. Plant Genome.

[bib59] Murali A., Bhargava A., Wright E.S. (2018). IDTAXA: a novel approach for accurate taxonomic classification of microbiome sequences. Microbiome.

[bib60] Muthukumaran P., Thiyagarajan G., Arun Babu R., Lakshmi B.S. (2018). Raffinose from Costus speciosus attenuates lipid synthesis through modulation of PPARs/SREBP1c and improves insulin sensitivity through PI3K/AKT. Chem. Biol. Interact..

[bib61] Nagura T., Hachimura S., Hashiguchi M., Ueda Y., Kanno T., Kikuchi H., Sayama K., Kaminogawa S. (2002). Suppressive effect of dietary raffinose on T-helper 2 cell-mediated immunity. Br. J. Nutr..

[bib62] Nguyen S.G., Kim J., Guevarra R.B., Lee J.-H., Kim E., Kim S.-I., Unno T. (2016). Laminarin favorably modulates gut microbiota in mice fed a high-fat diet. Food Funct..

[bib63] Oksanen J., Blanchet F.G., Friendly M., Kindt R., Legendre P., McGlinn D., Minchin P., O'Hara R., Simpson G., Solymos P., Stevens M., Szöcs E., Wagner H. (2019). Vegan: community ecology package. R package version.

[bib64] O'Sullivan D., Arora T., Durif C., Uriot O., Brun M., Riu M., Foguet-Romero E., Samarra I., Domingo-Almenara X., Gahan C.G.M., Etienne-Mesmin L., Blanquet-Diot S. (2024). Impact of western diet on enterohemorrhagic Escherichia coli colonization in the human in vitro mucosal artificial colon as mediated by gut microbiota. Nutrients.

[bib65] Paone P., Suriano F., Jian C., Korpela K., Delzenne N.M., Van Hul M., Salonen A., Cani P.D. (2022). Prebiotic oligofructose protects against high-fat diet-induced obesity by changing the gut microbiota, intestinal mucus production, glycosylation and secretion. Gut Microb..

[bib66] Parks D.H., Chuvochina M., Rinke C., Mussig A.J., Chaumeil P.-A., Hugenholtz P. (2022). GTDB: an ongoing census of bacterial and archaeal diversity through a phylogenetically consistent, rank normalized and complete genome-based taxonomy. Nucleic Acids Res..

[bib67] Peters B.A., Shapiro J.A., Church T.R., Miller G., Trinh-Shevrin C., Yuen E., Friedlander C., Hayes R.B., Ahn J. (2018). A taxonomic signature of obesity in a large study of American adults. Sci. Rep..

[bib68] Qin Y.-Q., Wang L.-Y., Yang X.-Y., Xu Y.-J., Fan G., Fan Y.-G., Ren J.-N., An Q., Li X. (2023). Inulin: properties and health benefits. Food Funct..

[bib69] Quast C., Pruesse E., Yilmaz P., Gerken J., Schweer T., Yarza P., Peplies J., Glöckner F.O. (2013). The SILVA ribosomal RNA gene database project: improved data processing and web-based tools. Nucleic Acids Res..

[bib70] Rahat-Rozenbloom S., Fernandes J., Gloor G.B., Wolever T.M.S. (2014). Evidence for greater production of colonic short-chain fatty acids in overweight than lean humans. Int. J. Obes..

[bib71] Rattigan R., Sweeney T., Maher S., Thornton K., Rajauria G., O'Doherty J.V. (2020). Laminarin-rich extract improves growth performance, small intestinal morphology, gene expression of nutrient transporters and the large intestinal microbial composition of piglets during the critical post-weaning period. Br. J. Nutr..

[bib72] Russell J.B. (2015). Bergeys Man. Syst. Archaea Bact..

[bib73] Schliep K.P. (2011). phangorn: phylogenetic analysis in R. Bioinformatics.

[bib74] Schneeberger M., Everard A., Gómez-Valadés A.G., Matamoros S., Ramírez S., Delzenne N.M., Gomis R., Claret M., Cani P.D. (2015). Akkermansia muciniphila inversely correlates with the onset of inflammation, altered adipose tissue metabolism and metabolic disorders during obesity in mice. Sci. Rep..

[bib75] Schwiertz A., Taras D., Schäfer K., Beijer S., Bos N.A., Donus C., Hardt P.D. (2010). Microbiota and SCFA in lean and overweight healthy subjects. Obes. Silver Spring Md.

[bib76] Seong H., Bae J.-H., Seo J.S., Kim S.-A., Kim T.-J., Han N.S. (2019). Comparative analysis of prebiotic effects of seaweed polysaccharides laminaran, porphyran, and ulvan using *in vitro* human fecal fermentation. J. Funct.Foods.

[bib77] Shakappa D., Talari A., Rajkumar H., Shujauddin M. (2018). Hypolipidemic effect of red gram. Cajanus cajan L.) Prebiotic Oligosaccharides in Wistar NIN Rats, J. Diet. Suppl..

[bib78] Shannon E., Conlon M., Hayes M. (2021). Seaweed components as potential modulators of the gut microbiota. Mar. Drugs.

[bib79] Singh V., Yeoh B.S., Chassaing B., Xiao X., Saha P., Aguilera Olvera R., Lapek J.D., Zhang L., Wang W.-B., Hao S., Flythe M.D., Gonzalez D.J., Cani P.D., Conejo-Garcia J.R., Xiong N., Kennett M.J., Joe B., Patterson A.D., Gewirtz A.T., Vijay-Kumar M. (2018). Dysregulated microbial fermentation of soluble fiber induces cholestatic liver cancer. Cell.

[bib80] Smith A.G., O'Doherty J.V., Reilly P., Ryan M.T., Bahar B., Sweeney T. (2011). The effects of laminarin derived from Laminaria digitata on measurements of gut health: selected bacterial populations, intestinal fermentation, mucin gene expression and cytokine gene expression in the pig. Br. J. Nutr..

[bib81] Song K., Xu L., Zhang W., Cai Y., Jang B., Oh J., Jin J.-O. (2017). Laminarin promotes anti-cancer immunity by the maturation of dendritic cells. Oncotarget.

[bib82] Stolfi C., Pacifico T., Monteleone G., Laudisi F. (2023). Impact of western diet and ultra-processed food on the intestinal mucus barrier. Biomedicines.

[bib83] Strain C.R., Collins K.C., Naughton V., McSorley E.M., Stanton C., Smyth T.J., Soler-Vila A., Rea M.C., Ross P.R., Cherry P., Allsopp P.J. (2020). Effects of a polysaccharide-rich extract derived from Irish-sourced Laminaria digitata on the composition and metabolic activity of the human gut microbiota using an in vitro colonic model. Eur. J. Nutr..

[bib84] Suriano F., Nyström E.E.L., Sergi D., Gustafsson J.K. (2022). Diet, microbiota, and the mucus layer: the guardians of our health. Front. Immunol..

[bib85] Takada T., Watanabe K., Makino H., Kushiro A. (2016). Reclassification of Eubacterium desmolans as Butyricicoccus desmolans comb. nov., and description of Butyricicoccus faecihominis sp. nov., a butyrate-producing bacterium from human faeces. Int. J. Syst. Evol. Microbiol..

[bib86] Theil S., Rifa E. (2021). rANOMALY: AmplicoN wOrkflow for Microbial community AnaLYsis. F1000Research.

[bib87] Thursby E., Juge N. (2017). Introduction to the human gut microbiota. Biochem. J..

[bib88] Van den Abbeele P., Grootaert C., Marzorati M., Possemiers S., Verstraete W., Gérard P., Rabot S., Bruneau A., El Aidy S., Derrien M., Zoetendal E., Kleerebezem M., Smidt H., Van de Wiele T. (2010). Microbial community development in a dynamic gut model is reproducible, colon region specific, and selective for Bacteroidetes and Clostridium cluster IX. Appl. Environ. Microbiol..

[bib89] Vigors S., O'Doherty J.V., Rattigan R., McDonnell M.J., Rajauria G., Sweeney T. (2020). Effect of a laminarin rich macroalgal extract on the caecal and colonic microbiota in the post-weaned pig. Mar. Drugs.

[bib90] Wang Y.-C., Ku W.-C., Liu C.-Y., Cheng Y.-C., Chien C.-C., Chang K.-W., Huang C.-J. (2021). Supplementation of probiotic Butyricicoccus pullicaecorum mediates anticancer effect on bladder urothelial cells by regulating butyrate-responsive molecular signatures. Diagn. Basel Switz..

[bib91] Watanabe H., Sonoyama K., Watanabe J., Yamaguchi N., Kikuchi H., Nagura T., Aritsuka T., Fukumoto K., Kasai T. (2004). Reduction of allergic airway eosinophilia by dietary raffinose in Brown Norway rats. Br. J. Nutr..

[bib92] Waters J.L., Ley R.E. (2019). The human gut bacteria Christensenellaceae are widespread, heritable, and associated with health. BMC Biol..

[bib93] Wu F., Guo X., Zhang J., Zhang M., Ou Z., Peng Y. (2017). Phascolarctobacterium faecium abundant colonization in human gastrointestinal tract. Exp. Ther. Med..

[bib94] Yassour M., Lim M.Y., Yun H.S., Tickle T.L., Sung J., Song Y.-M., Lee K., Franzosa E.A., Morgan X.C., Gevers D., Lander E.S., Xavier R.J., Birren B.W., Ko G., Huttenhower C. (2016). Sub-clinical detection of gut microbial biomarkers of obesity and type 2 diabetes. Genome Med..

[bib95] Yu Y., Lee C., Kim J., Hwang S. (2005). Group-specific primer and probe sets to detect methanogenic communities using quantitative real-time polymerase chain reaction. Biotechnol. Bioeng..

[bib96] Zhang P. (2022). Influence of foods and nutrition on the gut microbiome and implications for intestinal health. Int. J. Mol. Sci..

[bib97] Zhang M., Yang X.-J. (2016). Effects of a high fat diet on intestinal microbiota and gastrointestinal diseases. World J. Gastroenterol..

[bib98] Zhang P., Jiang G., Ma C., Wang Y., Yan E., He L., Guo J., Zhang X., Yin J. (2023). Dietary supplementation of laminarin improves the reproductive performance of sows and the growth of suckling piglets. J. Anim. Sci. Biotechnol..

