# Supplementary material for: Effects of prebiotics from diverse sources on dysbiotic gut microbiota associated to western diet: Insights from the human Mucosal ARtificial COLon (M-ARCOL)
Source: Curr Res Food Sci. 2024 Dec 26;10:100968. doi: 10.1016/j.crfs.2024.100968 (PMC11743849; doi:10.1016/j.crfs.2024.100968)
Supplement: Multimedia component 1 [file mmc1.docx]

*Original article, Current Research in Food Science*

**Effects of prebiotics from diverse sources on dysbiotic gut microbiota associated to western diet: insights from the human Mucosal ARtificial COLon (M-ARCOL)**

Ophélie Uriot^1^, Clémence Defois-Fraysse^2^, Ingrid Couturier^1^, Charlotte Deschamps^1^, Claude Durif^1^, Cyril Chaudemanche^3^, Assia Dreux-Zigha^2^, Stéphanie Blanquet-Diot^1,^*

**Supplementary figures:**

**Fig. S1:** **Effect of donors and colonic compartment on bacterial beta-diversity**

Beta-diversity of samples collected from the M-ARCOL was assessed by PCoA highlighting significant donor effect (**a**). Redundancy analysis (RDA) two-dimension plot visualization of bacterial diversity also showed the impact of colonic microenvironments when removing donor effect (**b**) and donor effect for each experimental condition (**c**). HD: healthy diet, INU: inulin, LAM: laminarin, RFO: raffinose family oligosaccharides, WD: western diet.

**
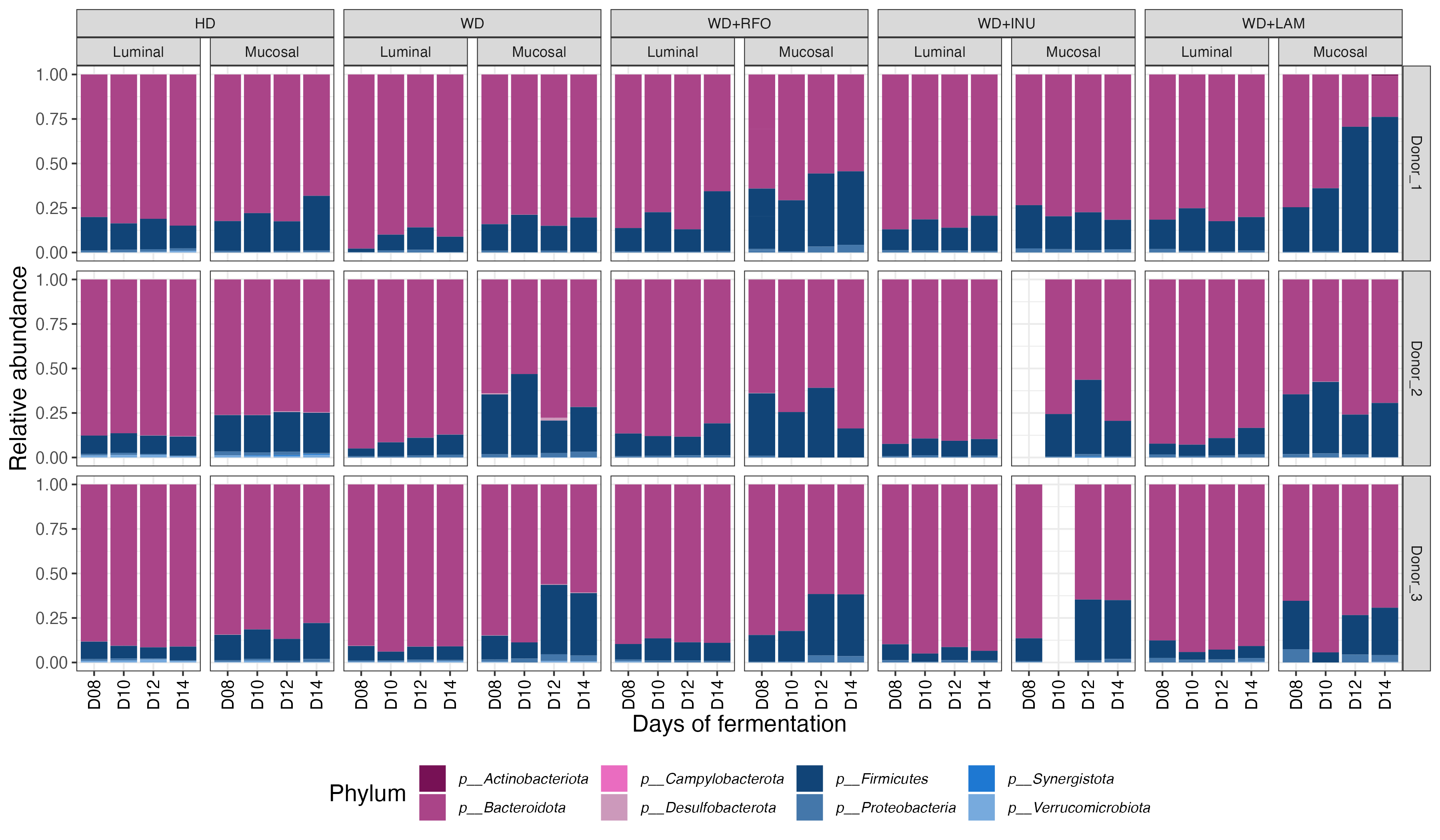
**

**Fig. S2 Impact of prebiotic treatments on microbial composition in the M-ARCOL at the phylum level**

Lumen and mucus-associated microbiota composition was analysed by 16S rRNA Metabarcoding. Relative abundance of the main bacterial populations in both colonic microenvironments are represented only for stabilised days (days 8 to 14) at the phylum level. HD: healthy diet, INU: inulin, LAM: laminarin, RFO: raffinose family oligosaccharides, WD: western diet.

**Fig. S3: Differential analysis of the impact of different prebiotic supplementation on microbiota composition at family and genus level**

Lumen and mucus-associated microbiota were analysed by 16S rRNA Metabarcoding. Differential analyses were further performed on even days 8 to 14 (with the results from all three donors pooled) to highlight differentially expressed family and genus between prebiotic treatments under western diet condition. Differential analyses based on DESeq2, metagenomeSeq and metacoder (p<0.05) methods were performed. The solid histogram corresponds to luminal samples and hatched histogram corresponds to mucosal samples. Blue: WD+RFO, purple: WD+INU, orange: WD+LAM. INU: inulin, LAM: laminarin, RFO: raffinose family oligosaccharides, WD: western diet.
